# Thymosin β4 dynamics during chicken enteroid development

**DOI:** 10.1007/s11010-020-04008-x

**Published:** 2020-12-10

**Authors:** Mohan Acharya, Rohana Liyanage, Anamika Gupta, Komala Arsi, Ann M. Donoghue, Jackson O. Lay, Narayan C. Rath

**Affiliations:** 1 Department of Poultry Science, University of Arkansas, Fayetteville, AR 72701 USA; 2 Poultry Production and Product Safety Research, USDA/ARS, Fayetteville, AR 72701 USA; 3 Statewide Mass Spectrometry Facility, Department of Chemistry and Biochemistry, University of Arkansas, Fayetteville, AR 72701 USA

**Keywords:** Intestinal enteroids, Thymosin β4, Wound healing, Immunohistochemistry

## Abstract

The sheared avian intestinal villus-crypts exhibit high tendency to self-repair and develop enteroids in culture. Presuming that this transition process involves differential biomolecular changes, we employed matrix-assisted laser desorption ionization-time of flight mass spectrometry (MALDI-TOF–MS) to find whether there were differences in the spectral profiles of sheared villi versus the enteroids, assessed in the mass range of 2–18 kDa. The results showed substantial differences in the intensities of the spectral peaks, one particularly corresponding to the mass of 4963 Da, which was significantly low in the sheared villus-crypts compared with the enteroids. Based on our previous results with other avian tissues and further molecular characterization by LC-ESI-IT-TOF–MS, and multiple reaction monitoring (MRM), the peak was identified to be thymosin β4 (Tβ4), a ubiquitously occurring regulatory peptide implicated in wound healing process. The identity of the peptide was further confirmed by immunohistochemistry which showed it to be present in a very low levels in the sheared villi but replete in the enteroids. Since Tβ4 sequesters G-actin preventing its polymerization to F-actin, we compared the changes in F-actin by its immunohistochemical localization that showed no significant differences between the sheared villi and enteroids. We propose that depletion of Tβ4 likely precedes villous reparation process. The possible mechanism for the differences in Tβ4 profile in relation to the healing of the villus-crypts to developing enteroids is discussed.

## Introduction

The intestinal epithelium undergoes rapid turnover and renewal [1–4], a process that can potentially breach its barrier function making the intestine vulnerable to infection. Nonetheless, its rapid regenerative process maintains its homeostasis unscathed. The molecular basis of regenerative process in the intestine, however, is less understood. In the course of avian enterocyte culture, we observed that the intestinal mucosal crypt-villi exhibit high propensity to self-repair and generate spheroid like villus enteroids within 24 h in culture [5]. We hypothesized that this transition of sheared villi to enteroids may entail many biochemical changes in the tissues that may provide understanding into their regenerative process and identify biomarkers. MALDI-TOF–MS (matrix-assisted laser desorption ionization-time of flight mass spectrometry) has been a method to identify low mass biomolecules such as small proteins and peptides that are uniquely present in different cells, tissues, and bacteria, based both, on their ionization potential and abundance [6–12]. Previously, we identified differential presence of thymosin β4 (Tβ4) and different beta-defensins in avian macrophages, heterophils, and egg shell membranes [13–17]. Hence, it was of interest to find whether there were differential changes in MALDI-TOF–MS profiles of the enteroids when compared with the sheared villi, by screening the whole tissues or their extracts in the mass range of 2–18 kDa. The results of this study and their significance in relation to the villous enteroid formation is discussed.

## Materials and methods

### Tissue collection and the generation of enteroids

The villus-crypts enteroids were prepared using mucosal tissues harvested from the intestinal segment spanning between post pancreatic loop and ileocecal junction of day-old broiler chicks, euthanized by cervical dislocation [5]. The animal procedures were approved and conducted in accordance with the guidelines and regulations of the Institutional Animal Care and Use Committee of the University of Arkansas.

The intestinal segments from 5 to 6 birds each time, were gently milked longitudinally with flat-tip forceps to extrude mucosa into DMEM-F12 medium containing glutamine, HEPES, sodium bicarbonate (HiMedia Laboratories, LLC), sodium pyruvate, and antibiotic–antimycotic solutions (Sigma-Aldrich, Inc.), referred as complete medium, and dispersed by repeated pipetting. Those mucosal suspensions were centrifuged at 300×*g* for 10 min to remove supernatant. The pellets were reconstituted in fresh medium, and divided into 2 equal aliquots. One aliquot was passed through a 40 µm Falcon cell strainer (www.vwr.com), washed four times by successive transfer of the strainer through 3 petri dishes with complete medium to deplete single cells and debris where upon the filter retentate containing villus-crypts were collected for MALDI-MS and immunohistochemistry (IHC). The villus-crypts were concentrated by centrifugation at 300×*g* and the pellet was resuspended in a smaller volume of complete medium. For IHC, the villus-crypts were picked using a glass capillary under a dissecting microscope, and transferred to slides coated with Biobond (Electron Microscopy Sciences, www.emsdiasum.com). The rest of the villus-crypts were used for MALDI-MS and LC/MS–MS (liquid chromatography/tandem mass spectrometry) after concentrating the suspension by centrifugation. For villus enteroid culture, the other aliquot of mucosal tissues was centrifuged and the pellet reconstituted in the above complete medium supplemented with 10% HyClone fetal bovine serum (FBS; GE health Sciences, Logan, UT), 1 × bovine pituitary extract (BPE, Cell Applications, Inc., San Diego, CA), and insulin transferrin selenite (ITS, Sigma-Aldrich, Inc.) (culture medium), placed in hydrophobic 6 well culture plates (Sarstedt, Germany), and incubated overnight in CO_2_ incubator at 37 °C. Following incubation, the mucosal cultures were dispersed by gentle pipetting to remove attaching cells, and filtered through a 40 µm cell strainer as above to separate enteric spheroids from free cells and tissue debris by successive transfer and washing with excess volumes of complete medium as described above. Some enteroids were transferred to glass slides for IHC as described above and the rest concentrated by centrifugation at 300×*g* for 10 min and the pellets were processed for MALDI-MS and LC–MS/MS.

### MALDI-TOF–MS and statistical analysis

Approximately 20–30 villi pieces or enteric spheroids in complete medium were transferred to microtubes and centrifuged at 1000×*g* to remove the fluid and the pellets were mixed with 10 µl of 70% methanol containing 1% acetic acid. One to two µl of cell suspension were spotted and smeared on a Bruker MTP 384 stainless steel target and were overlaid with 1 µl of α-cyano-4-hydroxycinnamic acid (HCCA) matrix solution (40 mg HCCA/ml in 50% acetonitrile, 47.5% water and 2.5% trifluoroacetic acid acid), and subjected to MALDI-TOF–MS in the m/z range of 2–18 kDa [18]. Protein calibration standard (Bruker Daltonics), prepared similarly, was placed in adjacent spots and the spectra were acquired using a Bruker Reflex III MALDI-TOF mass spectrometer (Bruker Daltonik GMBH, Bremen, Germany) to calibrate time of flight. MALDI-MS spectra were obtained in positive linear ion mode using thousand laser shots accumulated to represent each sample. This process was repeated for at least five biological samples. Spectral data were processed using ClinProTools software version 2.2 (Bruker Daltonik, Germany). The calculation of peaks was based on a signal to noise threshold of 10 with 10% relative threshold base peak of total average spectrum obtained from five MALDI-TOF–MS spectra from five biological samples. Spectra were internally normalized before peak areas were calculated. Values with *P ≤ *0.05, calculated using student t-test, were considered significant.

### LC–MS and targeted MRM LC–MS/MS analysis of thymosin β4 (Tβ4)

Peaks observed in MALDI-TOF–MS were identified using exact intact mass information from liquid chromatography-electro spray ionization quadrupole ion trap-time of flight mass spectrometer (LC-IT-TOF–MS) and sequence specific fragment ions from LC–MS/MS. A HPLC-20A/LC-30A, coupled to a Shimadzu electro spray ionization quadrupole ion trap-time of flight mass spectrometer (LC-ESI-IT-TOF–MS) was used to obtain high resolution, accurate intact masses with less than 20 ppm error. Seventy percent methanol extracts of freshly harvested pellets of villi and enteroids were subjected to LC separations using a Bio wide Pore C18 reverse phase column (4.6 mm × 15 cm, 5 μm) (Supelco, St. Louis, MO) using 0.1% formic acid/acetonitrile gradient of 5–100% with a solvent flow rate of 0.8 ml/min over a 60 min period. Shimadzu LC–MS solution version 3.81 was used to process peaks to obtain mass spectra for each chromatographic peak. The isotopically resolved high resolution multiple charged ions were deconvoluted and the average of the isotopic masses weighted by isotopic abundances to obtain average neutral mass. Average neutral mass and a mass error of ± 20 ppm with variable protein N-terminal acetyl modification was searched in Uniprot Gallus protein data base using Mascot sequence query web tool in MASCOT software (http://www.matrixscience.com). Protein hits were filtered out assuming the observed peak corresponds to an intact peptide or a fragment of protein that starts before and after methionine in the list of peptide/proteins in order for identification. Based on the result as well as the prior knowledge that the m/z 4963 may be chicken Tβ4, structurally similar to its mammalian counterpart [13, 18], we validated its identity of this using a recombinant Tβ4 standard (www.peprotech.com) and multiple reaction monitoring (MRM) as follows. The targeted analysis of Tβ4 tryptic digests was performed using a Shimadzu UPLC-20A/LC-30A in line with the Shimadzu 8060 triple quadrupole mass spectrometer with a heated electrospray source (positive-ion mode). LC separations were performed using a C18 column (2.1 × 50 mm, 1.9 μm particle size, Shimadzu, UHPLC check out kit) with a linear gradient composed of 0.1% formic acid (FA) in acetonitrile, ramped at a rate of 7.5% acetonitrile/min over 10 min. The flow rate was 0.3 ml/min. Prior to LC–MS/MS analysis, 70% ethanol extract of enteroids, and about 5 µg of Tβ4 standard were subjected to trypsin digestion separately. The extracts were dried with a Speedvac concentrator (Labonco), reconstituted with 100 µl of 25 mM ammonium bicarbonate, and digested with 1 µg of MS grade trypsin (www.Promega.com) prepared in 50 µl of 25 mM ammonium bicarbonate, and incubated overnight at 37 °C. One hundred µl of 5% FA in 60% acetonitrile was added to quench trypsin and break down ammonium bicarbonate. Digest mixtures were then dried as above and reconstituted in 50 µl of 0.1% FA and proceeded to optimize MRM transitions for the tryptic peptides from Tβ4 standard. The peak at m/z 4963 was confirmed to be Tβ4 by targeting its tryptic peptides, SDKPDMAEIEK (acetylate N-terminus), NPLPSK, and ETIEQEK, to LC–MS/MS using a Shimadzu 8050 liquid chromatography triple quadrupole mass spectrometer. Optimized MRM transitions of SDKPDMAEIEK (N-terminus acetylated): (652.8^++^ 720.4^+^[y6], 787.3^+^[b7], 916.4^+^[b8], 932.4^+^ [y8], 835.4^+^[y7]), NPLPSK: (328.2^++^ 325.2^+^[b3], 541.3^+^[y5], 444.3^+^[y4], 331.2^+^[y3]), and ETIEQEK: (438.7^++^ 747.4^+^[y6], 646.3^+^[y5], 533.3^+^ [y4]) of the respective tryptic peptides from the standard Tβ4, were used as the reference. Intensity ratios MRM transitions and the retention times from the tryptic peptides and intact mass of the standard Tβ4 were compared with the peptides obtained from the extracts of enteroid samples.

### Immunofluorescence localization of Tβ4 and F-actin

The slides containing sheared villi crypts and the enteroid samples were wetted with phosphate buffered saline (PBS) then treated with Cytovista 3D clearing agent (www.thermofisher.com) for 10 min followed by an additional washing step with PBS. The tissues were then permeabilized with 0.5% Triton X-100, and blocked with 10% goat serum (Sigma-Aldrich, Inc.) in PBS for an hour. The tissues were rinsed with PBS once to remove the blocking solution and the excess PBS was removed by vacuum aspiration. A rabbit anti-Tβ4 antibody (https://www.abcam.com) prepared in PBS containing 0.1% bovine serum albumin (BSA) placed over the tissues and incubated overnight at 4 °C in a humidified chamber. The following day, the slides were rinsed 3 times with PBS and probed with a secondary goat anti-rabbit IgG conjugated to Alexa fluor 488 (Abcam) for 1 h at room temperature in dark. After 3 further wash steps with PBS, the tissues were counterstained with 4′, 6-diamidine-2′-phenylindole dihydrochloride (DAPI; 0.2 µg/ml PBS) and mounted with ProLong gold anti-fade reagent (www.thermofisher.com). F-actin was stained using Acti-stain (Alexa 550 labeled phalloidin, www.cytoskeleton.com) following the recommended protocol of the manufacturer. The images were photographed with an Olympus BX microscope equipped with fluorescent optics and Image Pro Premier software (http://www.mediacy.com/imagepro).

### Effect of disruption damage induced changes in enteroid Tβ4

To find whether structural damage to the enteroids affect changes in the Tβ4 activities, we used freshly filtered enteroids, divided into 2 aliquots each containing approximately 50 enteroids in 250 µl of complete medium. One of the aliquot was subjected to vigorous disruption using an orbital shaker set to a maximum speed and the other left intact. Both samples were then centrifuged at 300×*g* for 10 min. Care was taken not to disturb the pellet when supernatants were aspirated to dryness as much as possible. The pellets were then dissolved in 10 µl of 70% ethanol containing 1% acetic acid and one to two µl of cell suspension subjected to MALDI-TOF MS as described above. The assays were repeated two times on different days.

## Results

### Enteroids and the identification of Tβ4

**Fig. 1:**
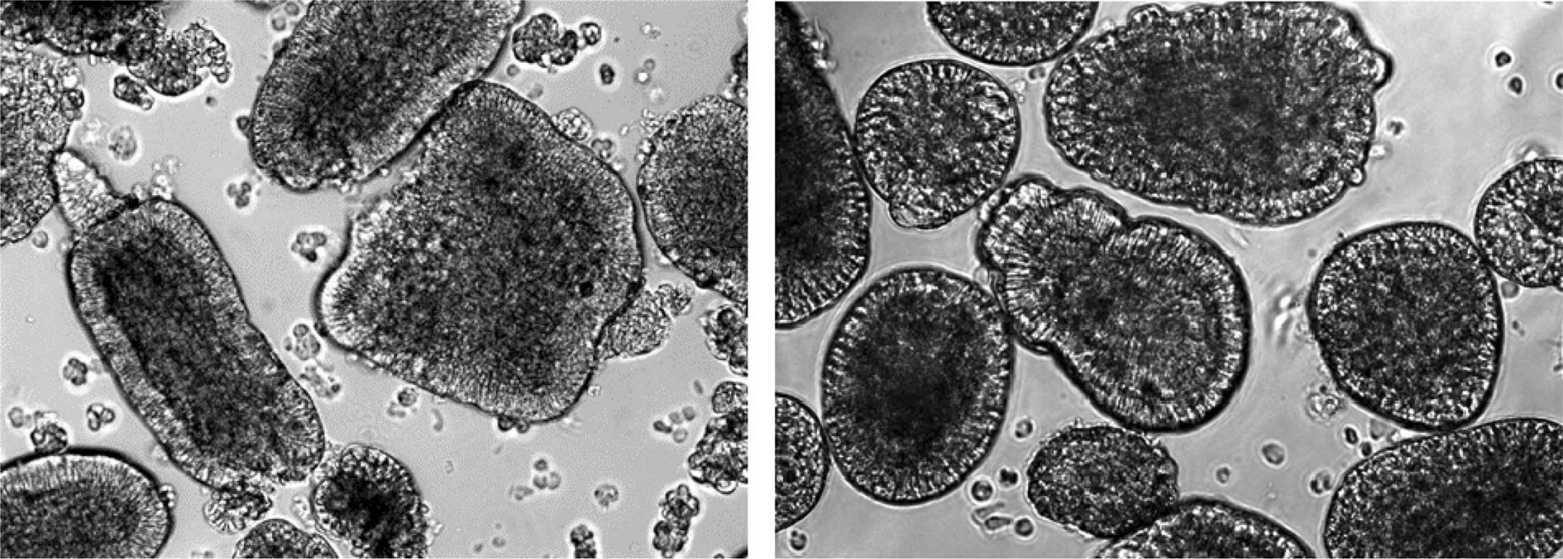
Sheared Villous-crypts following harvest (left) and after 20 h culture (right) following filtration through 40 µm filter (× 100 magnification)

**Fig. 2:**
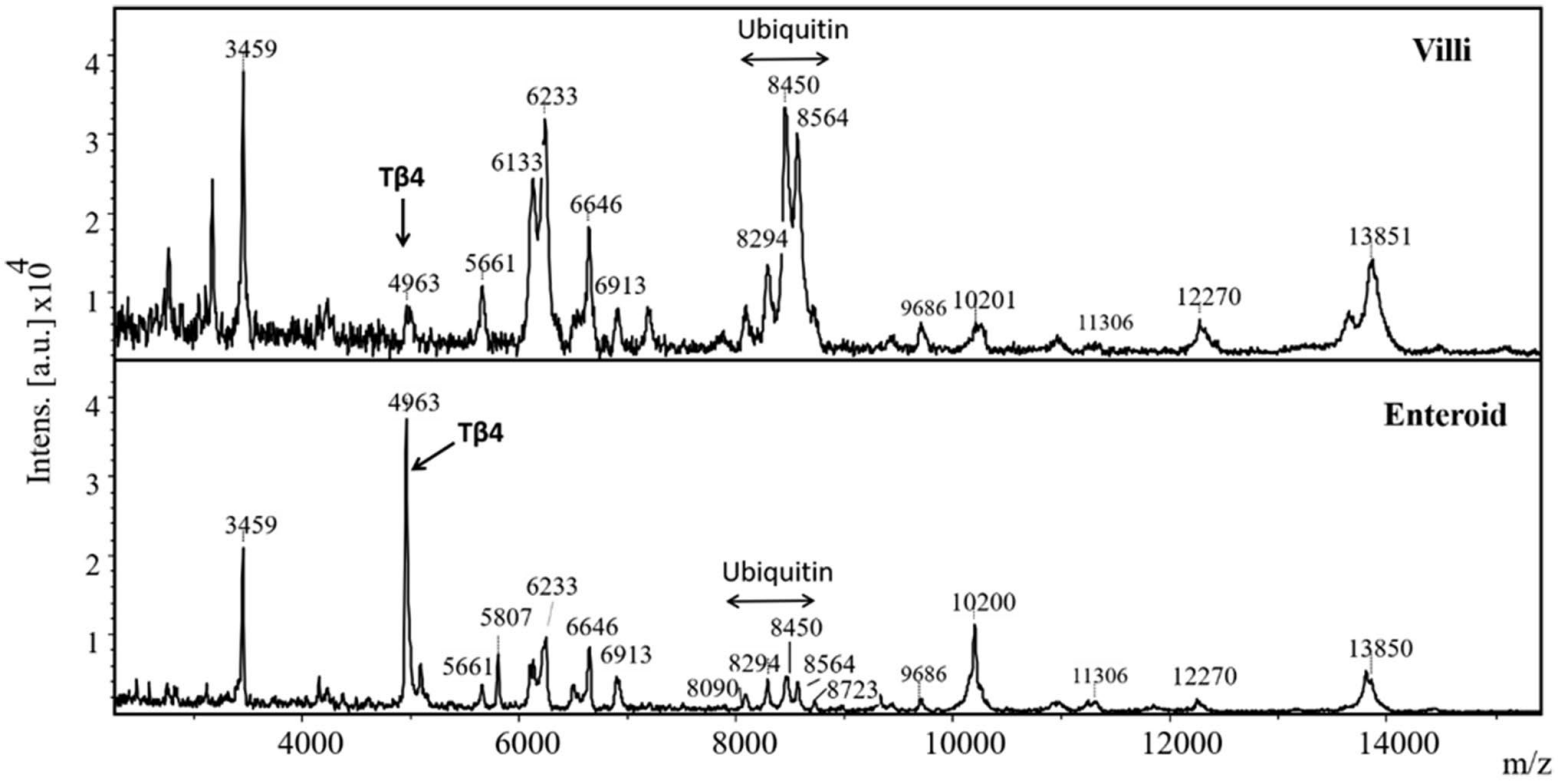
MALDI-TOF-mass spectrogram of enterocyte extract. The identity of the peaks were based NCBI blast search of the Uniprot identifications

**Fig. 3:**
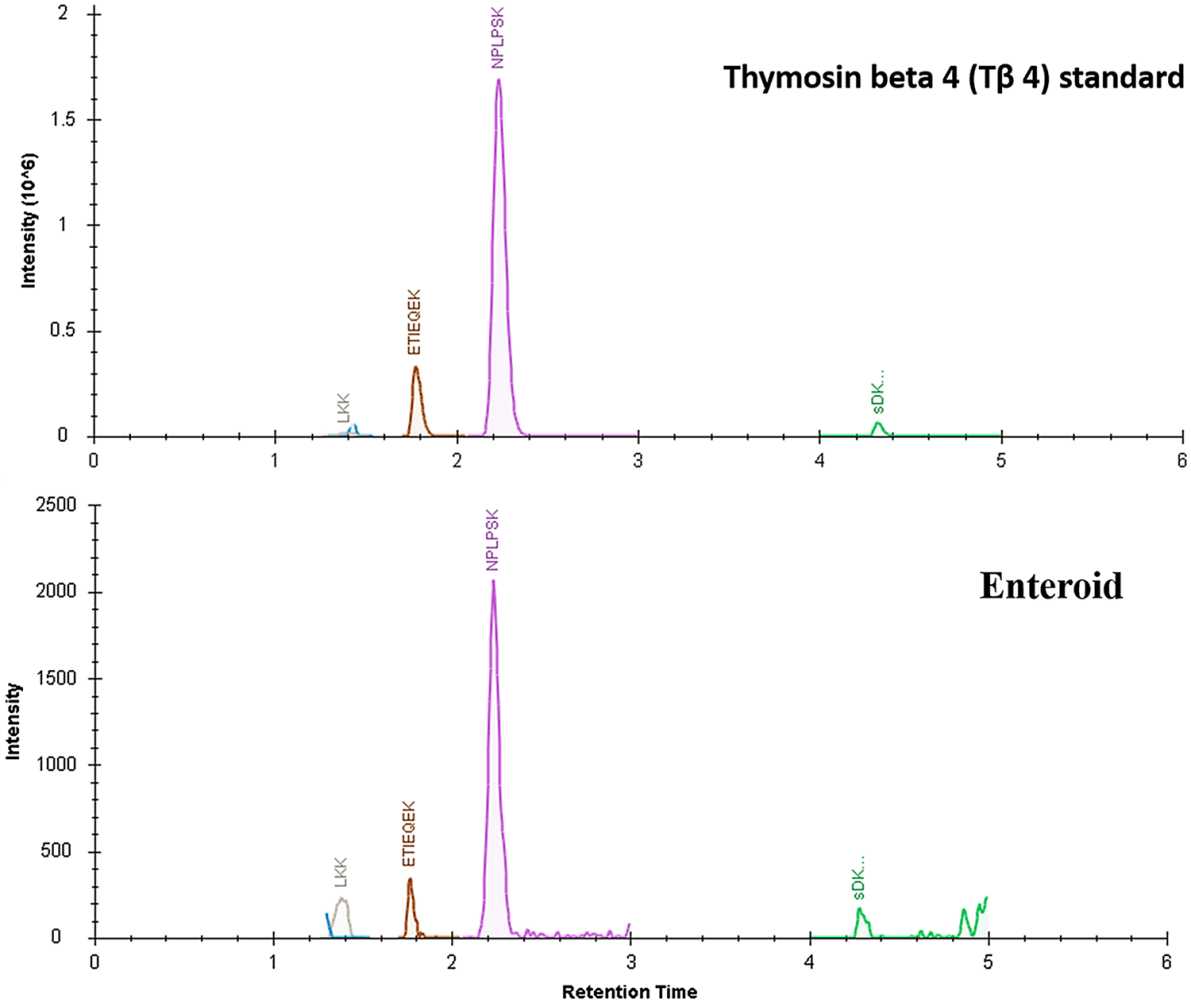
Targeted identification of Tβ4 peptides using standard Thymosin beta 4 tryptic digests (upper panel) and the enteroid extract treated similarly (lower panel). The peaks corresponding sum of MRM transitions of SDKPDMAEIEK (acetylate N-terminus), NPLPSK, ETIEQEK of Tβ4 and the corresponding enteroid peptides

Figure 1 shows the villi segments (left panel) and the enteroids obtained after filtrations (right panel). MALDI-TOF–MS profiles of both tissues were comparable (Fig. 2) although there were many differences with respect to relative spectral intensities as evident from Bruker ClinProTool statistical analyses (Table 1). Of the spectral peaks showing significant statistical differences, the most prominent one was a peak corresponding to m/z 4963. Based on our prior studies with chicken cells, employing MALDI-TOF–MS, the m/z 4963 was then known to be thymosin β4 (Tβ4) [13]; however, the identity of Tβ4 in the current study was further confirmed using exact neutral mass 4963.419 obtained from IT-TOF–MS, and a MASCOT sequence query and by multiple reaction monitoring (MRM). The MRM transitions for the peptides derived from a recombinant Tβ4 standard based on the retention times and the relative intensities of its peptides, SDKPDMAEIEK, NPLPSK, and ETIEQEK to validate corresponding enteroid Tβ4 (Fig. 3). Some of the other MALDI-TOF–MS peaks were also tentatively identified using IT-TOF–MS derived average neutral mass values shown it Table 1 and Mascot sequence query. The peaks at m/z 8294, 8450 and 8564 were identified as ubiquitin or its fragments. The other peaks, m/z 3459, 5661, 5807, 6133, 8723, 10,200, 11,306, 13,851, were identified as phosphoenolpyruvate carboxykinase, POU homeodomain protein, MHC class 1 antigen, cardiac phospholamban, MLLT11 transcription factor 7 cofactor (protein AF1q), epidermal differentiation protein, C-type lectin domain family 2 member B, and platelet glycoprotein VI-like, respectively (Fig. 2, Table 1). The common names of these protein were obtained using protein NCBI blast search of the Uniprot identifications in Gallus gallus (taxid: 9031).

**Table 1:** ClinProTool statistical analyses and tentative identification of MALDI-TOF mass spectra peaks

Mass	Average neutral mass (IT-TOF)	Protein	DAve	*P* (*t*-test)	Ave1 (Villi)	Ave2 (Enteroid)	Std, Dev. (Villi)	Std, Dev. (Enteroid)	CV1 (Villi)	CV2 (Enteroid)
3459		Phosphoenolpyruvate carboxykinase Q6WQI7 CHICK	13	0.5	197	184	21	53	10	29
4643	4963.419	Thymosin β4 Q6WEB3_CHICK	697	< 0.0001	55	752	5	68	9	9
5661	5661.442	POU homeodomain protein A0A1D5PXA9_CHICK	46	< 0.0001	123	76	21	3	17	4
5807	5807.467	MHC class1 antigen A0A140KEG3_CHICK	147	< 0.0001	19	166	3	12	2	7
6133	6133.478	Cardiac phospholamban PPLA_CH1CK	97	0.0003	315	217	51	27	16	12
6233			222	< 0.0001	441	219	80	40	18	18
6646			2	0.9	155	156	13	25	8	16
6913			59	< 0.0001	73	132	19	6	25	4
8090			35	< 0.0001	96	61	15	2	15	4
8294	8294.458	Ubiquitin A0A1L1RWB4_CHICK	65	< 0.0001	162	97	23	14	14	14
8450	8450.638	Ubiquitin A0A1L1RWB4_CHICK	334	< 0.0001	491	157	89	6	18	4
8564	8564.575	Ubiquitin A0A1L1RWB4_CHICK	313	< 0.0001	418	106	88	2	21	2
8723	8723.627	MLLT11 transcription factor 7 cofactor A0A1L1RVM4_CHICK	72	< 0.0001	108	30	24	1	23	4
9685	9685.755		3	0.4	63	60	8	5	13	8
16,200	10,200.052	Epidermal differentiation protein A0A088BHB9_CHICK	235	< 0.0061	63	298	7	23	11	8
11,306	11,305.662	C-type lectin domain family 2 member B A0A1L1RLM1_CHICK	54	< 0.0001	28	82	2	8	7	10
12,234			42	< 0.0001	50	92	10	1	20	2
13,792			56	0.0004	115	170	25	22	22	13
13,851	13,850.583	Platelet glycoprotein VI-like A0A1L1RRW8_CHICK	91	0.001	186	95	60	13	32	14

*Mass* nominal m/z values from MALDI-TOF–MS, *average neutral mass (IT-TOF–MS)* deconvoluted weighted average of the isotopic masses weighted by the isotopic abundances, *uniprot identification* tentative identification using exact mass measured using IT-TOF–MS, *Dave* difference between the maximal and the minimal average peak area/intensity of all classes, *P-(t-test) P*-value of the student *t*-test, *AveN* normalized peak area/intensity average of class of villi and enteroids, *StdDev1* standard deviation of the peak area/intensity average of class villi and enteroid, *CVN* coefficient of variation in % of villi and enteroid

**Fig. 4:**
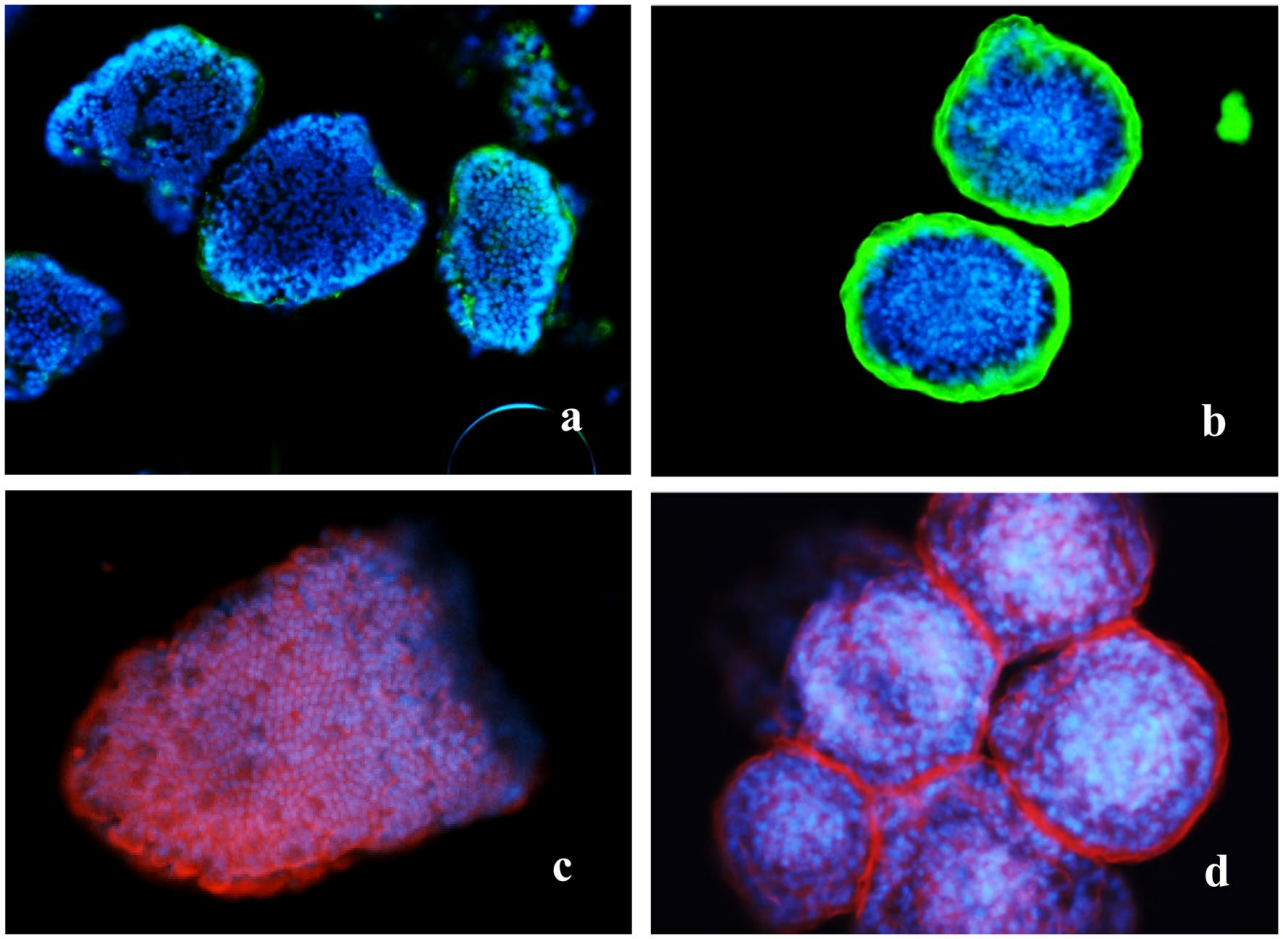
Immunochemical localization of thymosin β4 (**a**, **b**) and F-actin (**c**, **d**) in mucosal villi and enteroids, respectively. × 100 magnification

**Fig. 5:**
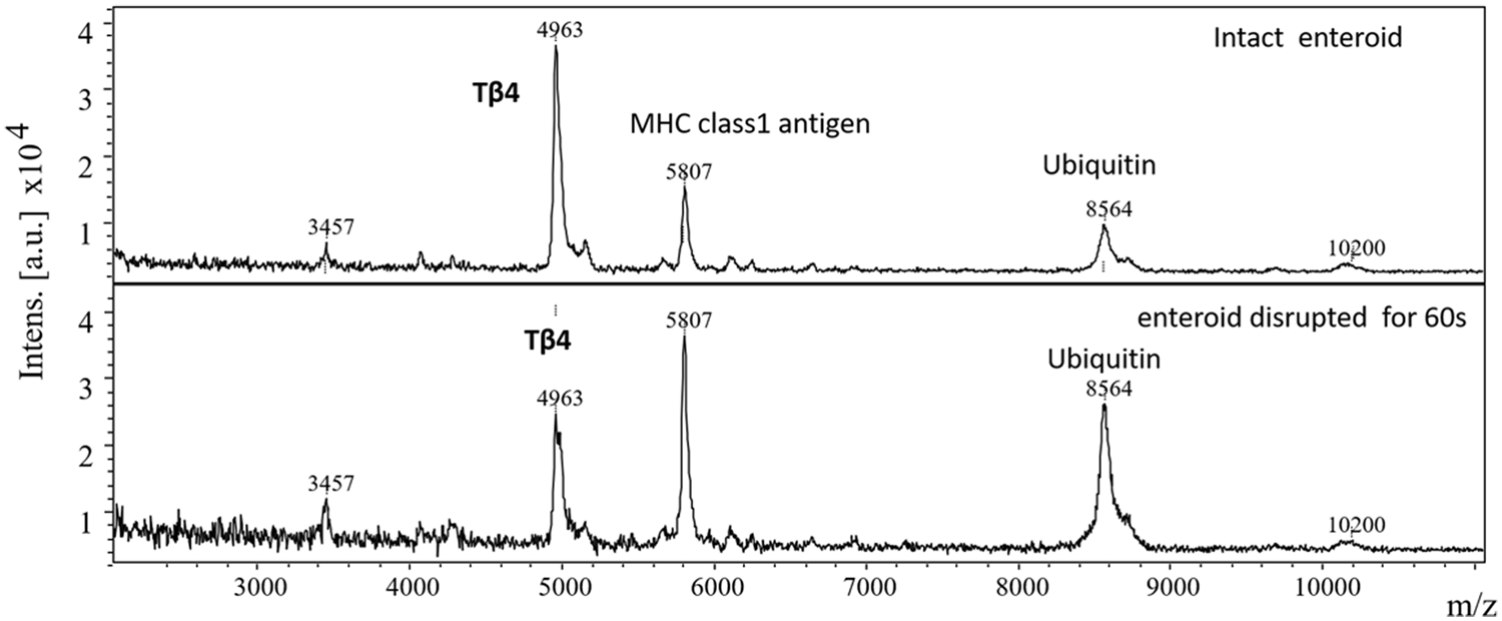
Effect of shaking induced damage on Tβ4 levels of enteroid extracts. (**a**) Intact enteroids and (**b**) enteroids subjected to vigorous shaking induced damage for 60 s

IHC of the villous crypts and the enteroids showed the later having significantly higher densities antibody reactive Tβ4 (Fig. 4a, b). The same trend was not evident with F-actin which was probed using alexa fluor 450 bound phalloidin and showed similar staining intensities in villi and the enteroids (Fig. 4c, d). Shaking induced damage reduced the spectral intensities of Tβ4 in relation to both other peaks, m/z 5807 and ubiquitin, m/z 8564 (Fig. 5).

## Discussion

From the results, it is evident that the sheared villi and the enteroids shared comparatively similar molecular profiles although they were largely different in their peak intensities. Some of these peaks were tentatively identified and we presume that their changes may be of biological significance; however, we were puzzled by the changes in the levels of Tβ4, a peptide that has been implicated in many biological functions particularly associated with wound healing [19]. Tβ4 was depleted in the sheared villi while it appeared replete in the enteroids which was also evident from the immunohistochemical staining of the respective tissues. Tβ4 promotes wound healing in cornea [20–22], dermis [23, 24], and cardiac tissues [25] although the exact mechanisms of its action are not well understood. Tβ4 as such has been implicated in many different biological functions which range from its ability to support cell survival, migration, differentiation to anti-inflammatory and antioxidant activities [26, 27]. Experiments using Tβ4 gene silencing have shown mixed results; in some tumorigenic cell lines it promoted cell differentiation but suppressed their migration, invasiveness, and proliferation [28, 29] whereas, in hepatic stellate cells it was reported to promote cell proliferation [30]. The role of Tβ4 in the gut tissues is little known although its presence in the ileum and the enterocytes was reported by Nemolato et al. using immunohistochemical localization that showed its heterogeneous expression associated with different phases of development [31]. An experimental knock down of Tβ4 gene was reported to increase DNA re-replication in intestinal epithelial cells [32]. However, a well-studied function of Tβ4 is that, it regulates both polymerization and depolymerization of cytoskeletal actin and sequesters the monomeric G-actin, preventing its assembly into filamentous F-actin [33]. F-actin formation is essential for cellular mobility, protrusion, cytokinesis, and the maintenance of its polarity and cell junction through its interaction with cell membrane [34–36]. Thus, it is considered important in tissue morphogenesis. F-actin occurs as a highly organized bundle located at the apical aspect of the epithelial enterocytes [37, 38]. The remodeling of actin is an important aspect of wound healing process [39]. The intestinal epithelial upon injury need to heal rapidly so as to maintain an effective barrier against penetration of microbes. Hence, the process may necessitate a rapid availability of free monomeric actins (G-actin) which is normally present intracellularly and maintained as complexes with different actin binding proteins such as Tβ4, cofilin, gelsolin and many others [27, 36]. Compared with other actin binding proteins, the intracellular concentration of Tβ4 is high, up to as much as 0.5 mM [40]. Hence, a rapid mobilization of Tβ4 in the injured tissue, to free up monomeric actin appear logical as indicated by a depleted level of Tβ4 in the sheared villi. Transitional depletion of Tβ4 could then promote F-actin polymerization and facilitate cell motility, cell division, and restore cohesivity promoting the healing of sheared villi to form enteroids. In the enteroids the Tβ4 level is restituted to its normal as evidenced by both by MALDI-MS and IHC. However, a similar depletion of F-actin was not evident using IHC which appeared to show comparable staining intensities in both villi and the enteroids. This raises the question as to the fate of free Tβ4 in the cells or to their degradation. We presume that, in the severed villi the free Tβ4 is released into the wash medium because this peptide is highly soluble in aqueous media which is why it is high in wound fluids [41, 42]. Experimental damage to the enteroids showed a decrease in cellular Tβ4 levels in our current study. It is also likely that tissue damage causes a rapid degradation of Tβ4 by some mechanism as ubiquitination. Ubiquitination leads to protein degradation which mediates many aspects of cellular homeostasis such as cellular stress and inflammation [43, 44]. However, its role in the process of wound healing and regeneration is less known. We observed, a reciprocal increase in the levels of ubiquitin in relation to Tβ4 in sheared as compared with the enteroids.

In conclusion, our results show that Tβ4 expression in the intestinal villi may vary depending upon the physiological states of the mucosal epithelium. We propose that the depletion of Tβ4 in wounded mucosa is related to its release from the cells freeing G-actin and its depletion may be a necessary step to facilitate actin polymerization, an essential aspect of tissue repair.

## Data Availability

Data available on request.

## Abbreviations

DMEM-F12: Dulbecco’s modified minimum essential medium with Ham’s F-12 salt mixture
HCCA: α-Cyano-4-hydroxycinnamic acid
IHC: Immunohistochemistry
IT-TOF–MS: Ion trap-time of flight mass spectrometer
LC-ESI-IT-TOF–MS: Liquid chromatography-electro spray ionization quadrupole ion trap-time of flight mass spectrometer
MALDI-TOF–MS: Matrix-assisted laser desorption ionization-time of flight mass spectrometry
m/z: Mass/charge
MRM: Multiple reaction monitoring
Tβ4: Thymosin beta 4
